# EphA1 receptor tyrosine kinase is localized to the nucleus in rhabdomyosarcoma from multiple species

**DOI:** 10.1242/bio.059352

**Published:** 2022-10-10

**Authors:** Ronnie LaCombe, Alessandra Cecchini, Morgan Seibert, DDW Cornelison

**Affiliations:** ^1^ Division of Biological Sciences, University of Missouri, Columbia, MO 65211, USA; ^2^ Christopher S. Bond Life Sciences Center, University of Missouri, Columbia, MO 65211, USA

**Keywords:** Eph/ephrin, Protein localization, Rhabdomyosarcoma

## Abstract

While the typical role of receptor tyrosine kinases is to receive and transmit signals at the cell surface, in some cellular contexts (particularly transformed cells) they may also act as nuclear proteins. Aberrant nuclear localization of receptor tyrosine kinases associated with transformation often enhances the transformed phenotype (i.e. nuclear ErbBs promote tumor progression in breast cancer). Rhabdomyosarcoma (RMS), the most common soft tissue tumor in children, develops to resemble immature skeletal muscle and has been proposed to derive from muscle stem/progenitor cells (satellite cells). It is an aggressive cancer with a 5-year survival rate of 33% if it has metastasized. Eph receptor tyrosine kinases have been implicated in the development and progression of many other tumor types, but there are only two published studies of Ephs localizing to the nucleus of any cell type and to date no nuclear RTKs have been identified in RMS. In a screen for protein expression of Ephs in canine RMS primary tumors as well as mouse and human RMS cell lines, we noted strong expression of EphA1 in the nucleus of interphase cells in tumors from all three species. This localization pattern changes in dividing cells, with EphA1 localizing to the nucleus or the cytoplasm depending on the phase of the cell cycle. These data represent the first case of a nuclear RTK in RMS, and the first time that EphA1 has been detected in the nucleus of any cell type.

## Teaser

**Summary:** This paper shown the first example of a receptor tyrosine kinase localizing to the nucleus in a tumor cell, and one of the first examples of one of the Eph family doing so, in primary rhabdomyosarcoma tumors from dog and multiple RMS cell lines from mouse and human.

## INTRODUCTION

Receptor tyrosine kinases (RTKs) are typically single-pass transmembrane proteins responsible for responding to external stimuli by initiating intracellular signaling cascades and transmitting information about the external environment into the cell. Typically, RTKs form either homo- or heterodimers upon ligand binding, which promotes trans-autophosphorylation of the intracellular kinase domains and allows interactions with adaptor proteins and intracellular signal transduction machinery (Fantl et al., 1993; Hubbard and Till, 2000; Lemmon and Schlessinger, 2010). Following activation, RTKs are often endocytosed and trafficked to lysosomes for degradation or recycled and sent back to the cell surface (Sorkin and Von Zastrow, 2002). In addition to this canonical role, in some contexts RTKs can participate in intracellular signaling from organelles such as the mitochondria and the nucleus (Lin et al., 2001; Ni et al., 2001; Stephenson et al., 2012; Huo et al., 2014).

Of the 58 RTKs identified so far, 19 have been observed to localize to the nucleus (Robinson et al., 2000). This nuclear localization of RTKs is most often found in cancer, and with very few exceptions it is associated with a less favorable clinical prognosis (Huo et al., 2014). Of the RTKs which have been observed in the nucleus, the ErbB family are the best characterized. ErbB1, ErbB2, and ErbB3 have all been detected in the nucleus as full-length polypeptides and are associated with increased proliferation and decreased sensitivity to growth-inhibitory signals (Lo et al., 2005; Wang and Hung, 2009; Stephenson et al., 2012). ErbB4, on the other hand, is associated with growth suppression and better prognosis. Interestingly, it exists in the nucleus as a cleaved intracellular fragment instead of a full-length protein and when in the nucleus it causes growth inhibition and even apoptosis (Muraoka-Cook et al., 2008; Stephenson et al., 2012). It has been speculated that these opposing effects on tumor progression might arise from the differential mechanisms of membrane-to-nucleus trafficking in different family members (Carpenter and Liao, 2009).

Rhabdomyosarcoma (RMS) is an aggressive childhood cancer that accounts for nearly half of all childhood soft tissue tumors (Gurney et al., 1999). There are about 350 new cases a year, and in cases where the cancer has already spread to distant sites, the survival rates drop to below 33% (Ognjanovic et al., 2009). There are two main subtypes of RMS, which were named based on their distinctive cell morphologies – embryonal (ERMS) and alveolar (ARMS). ERMS cells resemble undifferentiated myoblasts or embryonic myotubes and accounts for 75% of RMS cases (Gurney et al., 1999; Caserto, 2013). It is the less-aggressive type with 5-year survival rates of 73.4% (Ognjanovic et al., 2009). ERMS typically has multiple and diverse somatic mutations, but most often includes loss of heterozygosity on chromosome 11, with preferential loss of the maternal allele. This region, 11p15, contains at least three tumor suppressor genes (*IGF2*, *CDKN1C*, and *H19*) (Loh et al., 1992; Barr and Womer, 2009). Other mutations that are often associated with ERMS occur in *PTCH1*, *TP53*, and genes encoding proteins involved in the Ras pathway (Barr and Womer, 2009). The morphology of ARMS cells is reminiscent of alveoli in the lungs; this tumor type is more aggressive, with 5-year survival rates of 47.8% (Ognjanovic et al., 2009). While the etiology of ERMS is unclear, ARMS tumors are often characterized by a translocation of chromosomes 2 or 1 and chromosome 13 that creates a novel functional transcription factor fusing the PAX3 or PAX7 DNA binding domain (respectively) to the FOXO1 transactivation domain, with few or no additional tumor-promoting genetic mutations (Galili et al., 1993; Shapiro et al., 1993; Linardic, 2008). 77% of ARMS cases are ‘fusion positive’, with the PAX3:FOXO1 fusion occurring more often than the PAX7:FOXO1 fusion (Sorensen et al., 2002). Expression of the fusion protein is thought to contribute to ARMS by promoting transcription of genes that stimulate proliferation, promote cell survival by avoiding apoptosis, and suppress terminal differentiation (Epstein et al., 1995; Bernasconi et al., 1996; Anderson et al., 2001). Treatment for both types of RMS depends on the degree to which the cancer has progressed, but most often involves surgery, chemotherapy, and radiation.

RMS is classified as a skeletal muscle tumor based on its histological appearance and expression of muscle regulatory transcription factors such as myogenin (Barr and Womer, 2009; Caserto, 2013). It is the only tumor proposed to derive from a skeletal muscle lineage (Abraham et al., 2014). Mature skeletal muscle is composed of terminally differentiated, multinucleated muscle fibers; these myonuclei have permanently exited the cell cycle and therefore cannot proliferate. Muscle growth, repair, or regeneration requiring the addition of new myonuclei requires the activity of a resident muscle-specific stem cell population (satellite cells) (Dumont et al., 2015; Sambasivan and Tajbakhsh, 2015; Forcina et al., 2019). These cells resemble myoblasts during development in that they retain the ability to proliferate, express stem cell markers such as Sca-1 and ABCG2, and express myoblast markers such as Pax7 and Pax3 (Seale et al., 2000; Kassar-Duchossoy et al., 2005; Mitchell et al., 2005; Tanaka et al., 2009). They also express the myogenic transcription factors MyoD and myogenin: while in normal muscle cells myogenin expression is limited to terminally differentiated cells that have permanently exited the cell cycle, proliferating RMS cells in tumors and in culture may express myogenin (Tapscott et al., 1993; Sebire and Malone, 2003).

The Eph receptor family is the largest RTK family in vertebrates. There are 14 members divided into two classes – EphA receptors (EphA1-8 and EphA10) and EphB receptors (EphB1-4 and EphB6) – which are categorized based on affinity for the two subclasses of membrane-bound ligands: ephrin-As and ephrin-Bs (Frisen et al., 1999). The ephrin-As (ephrin-A1-5) are tethered by a glycosylphosphatidlyinositol (GPI) anchor while ephrin-Bs (ephrin-B1-3) are transmembrane proteins (Pandey et al., 1995). Together, Ephs and ephrins constitute a bidirectional signaling system involved in contact-dependent cell-to-cell communication that can signal into the receptor (forward)- and ligand (reverse)-bearing cell or in both directions at the same time (Pasquale, 2010). Receptor–ligand binding is context dependent and highly promiscuous: the EphAs bind to most or all of the ephrin-As, and the EphBs bind to most or all of the ephrin-Bs (Frisen et al., 1999; Himanen et al., 2007).

As Ephs are the largest family of RTKs in humans and are expressed in most tissues throughout development as well as being highly represented in stem cell lineages, it is unsurprising that they have roles in a number of cancers including breast cancer, prostate cancer, lung cancer, and melanoma (Surawska et al., 2004). Their dysregulated expression can promote cancer formation, progression, and metastasis, or combinations thereof: Ephs and ephrins regulate tumor cell proliferation, invasion, epithelial-to-mesenchymal transition, angiogenesis, and renewal of cancer stem cells (reviewed in Surawska et al., 2004; Pasquale, 2010 and Chen, 2012). Some of these functions occur independent of ligand stimulation via Eph crosstalk with other transmembrane proteins (Chong et al., 2000; Kullander and Klein, 2002).

To begin to study potential roles for Ephs and ephrins in RMS, we performed an antibody screen on ARMS and ERMS cells from three mammalian species. While multiple Ephs and ephrins are expressed at different levels and in different subsets of cells, we were intrigued to note that one protein, EphA1, localized to the nuclei of canine, murine, and human RMS cells and did not show this expression in untransformed primary canine or murine cells. Western blotting on cell lysates suggests that nuclear EphA1 is the full-length receptor rather than a cleavage product. Further, nuclear EphA1 localization *in vitro* is dependent on the phase of the cell cycle. This is the first time that EphA1 has been detected in the nucleus of any cell type, and EphA1 is the first nuclear RTK to be detected in RMS. These data suggest a potential conserved role for EphA1 in RMS cells of both ARMS and ERMS origin.

## RESULTS

### Primary canine RMS tumors express multiple Ephs and ephrins

**Fig. 1.**
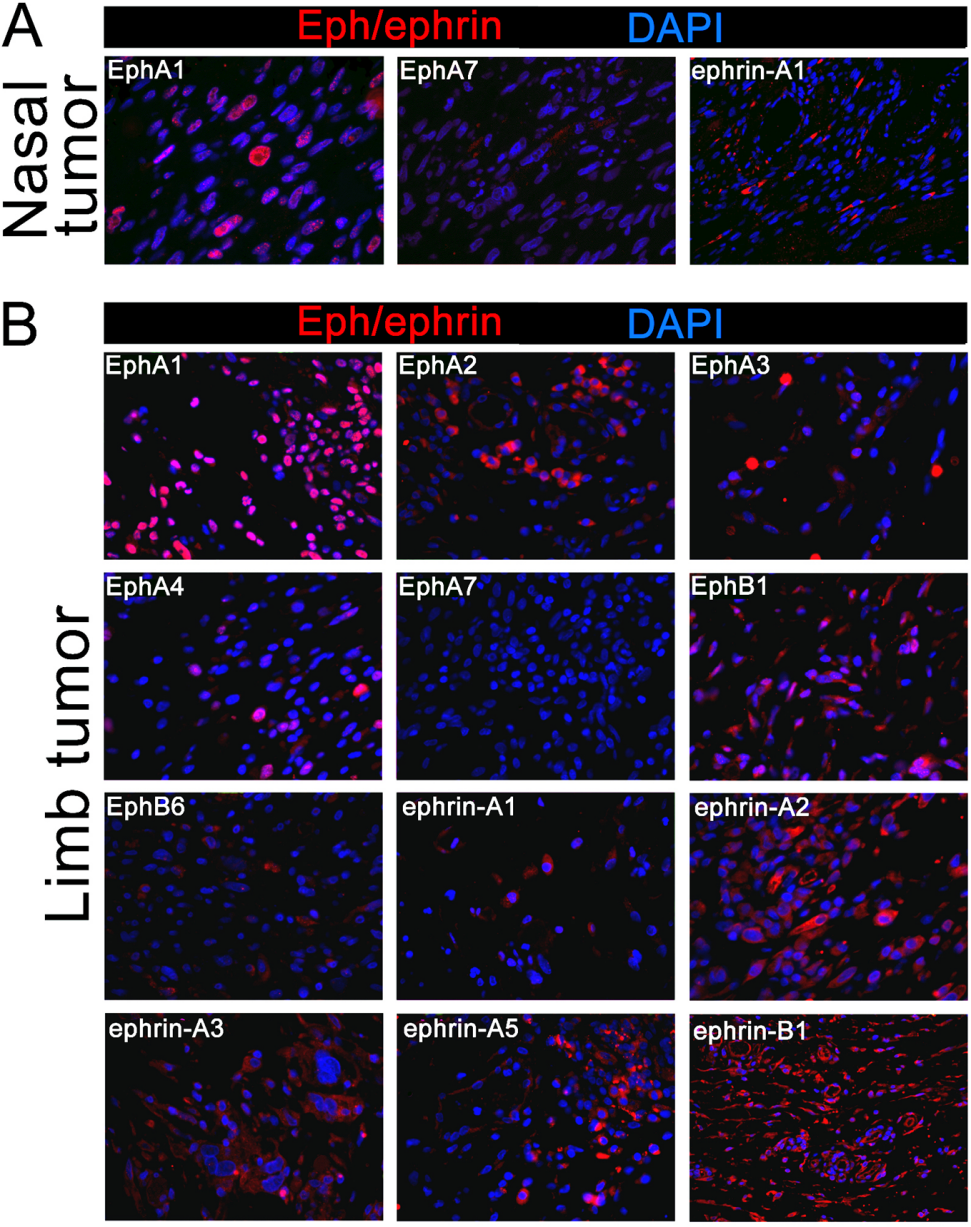
**Eph and ephrin protein expression screen in primary canine RMS tumors.** Eph and ephrin protein expression was surveyed by immunofluorescence in formalin fixed and paraffin embedded specimens of primary RMS from two different dogs located in the nasal cavity (A) and the limb (B). Sections were stained for Ephs and ephrins (Alexa 555, red) and costained with DAPI (blue) to visualize nuclei; Ephs and ephrins with expression above background in either specimen are shown. While expression of individual Ephs and ephrins was variable in intensity and in prevalence both within and between specimens, EphA1 was strongly expressed in the nucleus of cells of both tumors.

To assay Eph/ephrin expression in primary canine RMS specimens, we immunostained sections from two primary tumors identified as RMS by pathology after resection from two different companion dogs. One tumor was located in the nasal cavity and was surrounded by cartilage and mucosal tissue. The other was located in the limb tumor and was embedded in unaffected skeletal muscle. Both tumors were formalin fixed and paraffin embedded before sectioning. These sections were stained for Eph and ephrin proteins as well as for myogenin, a myogenic transcription factor that is commonly used as a RMS marker. Myogenin was not detectable in the limb tumor but was detected in a subset of cells in the nasal tumor; myogenin expression can vary widely between RMS tumors (Sebire and Malone, 2003). The nasal tumor was probed for EphA1, EphA3, EphA7, EphB4, ephrin-A1, and ephrin-B2. Expression of EphA1, EphA3, and ephrin-A1 was detected at varying levels (Fig. 1A). In the limb tumor, we detected EphA1, EphA2, EphB1, ephrin-A2, and ephrin-B1 at high levels, and EphA3, EphA4, EphB4, EphB6, ephrin-A1, ephrin-A3, ephrin-A4, and ephrinA5 at low levels, while EphA7, EphA8, EphB2, and ephrin-B2 were not detected (Fig. 1B). It is not unexpected that subsets of cells are positive for Ephs and ephrins as the tumor sections contain a heterogenous population of cells.

Most of the Ephs and ephrins detected displayed localization consistent with their status as membrane-localized proteins, with one notable exception. In both the nasal and limb tumors, EphA1 staining was consistently detected in the nucleus rather than at the plasma membrane, with nonuniform distribution (Fig. 1A).

### Eph and ephrin expression in mouse and human RMS cell lines

To leverage comparative biology between species, we compared Eph/ephrin expression in primary canine tumor specimens to mouse and human cell lines. Mouse RMS cell lines were derived from primary or metastatic tumors in transgenic mice designed to develop ERMS or ARMS: cell lines U23674 and U48484 represent the ARMS subtype as they are derived from mice with forced expression of the Pax3-FOXO1 fusion protein (Keller and Capecchi, 2005), while cell lines U33915 and U57810 represent the ERMS subtype, which does not express the fusion protein but instead is driven by mutations to *PTCH1* and *p53* in a muscle-specific fashion (Rubin et al., 2011).

**Fig. 2.**
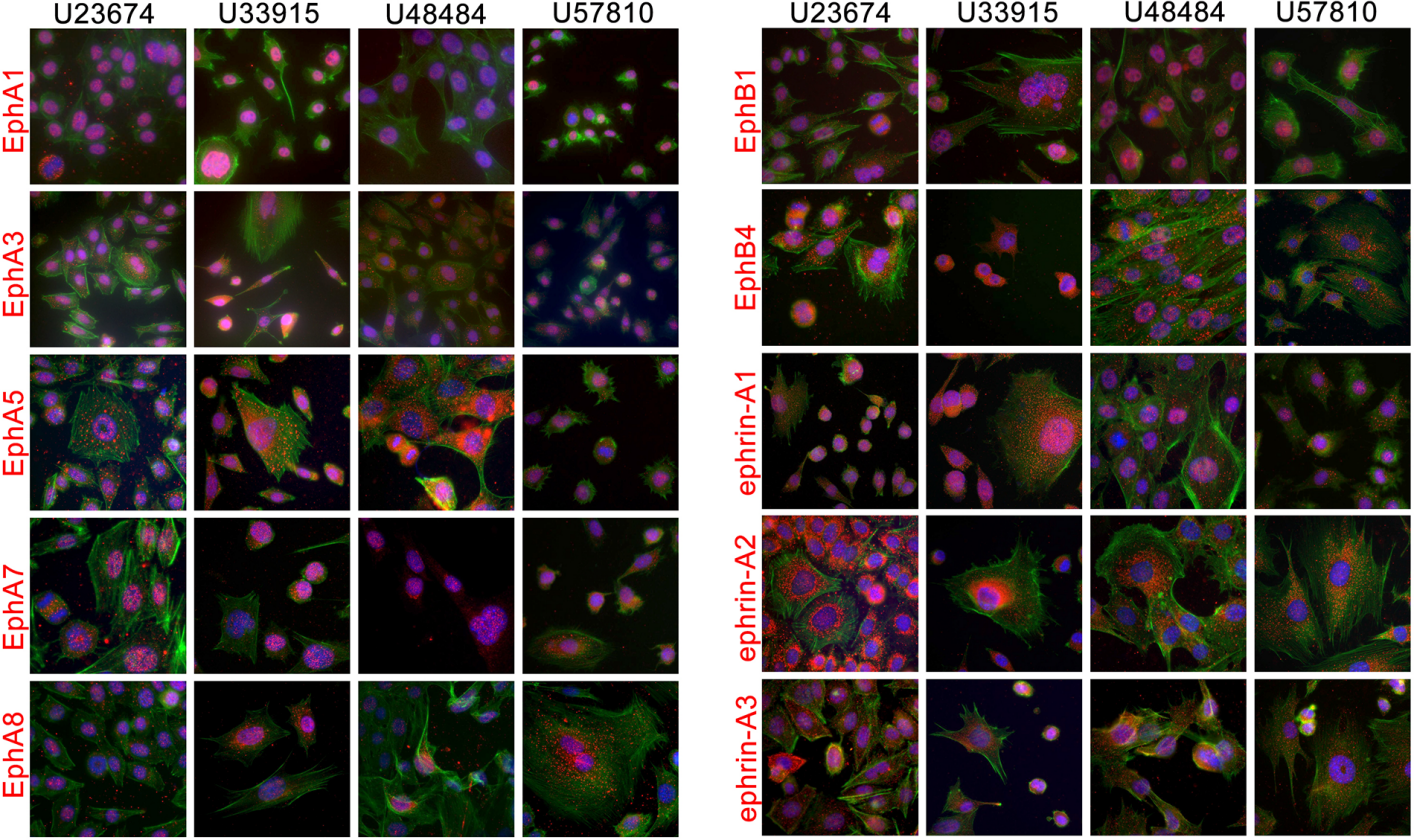
**Eph and ephrin protein expression screen in murine RMS cell lines.** Eph and ephrin protein expression was surveyed by immunofluorescence in four primary murine RMS cell lines: U23674 (ARMS), U33915 (ERMS), U48484 (ARMS), U57810 (ERMS). Cells were stained for individual Ephs and ephrins (Alexa 555, red) and costained with phalloidin (Alexa 488, green) and DAPI (blue) to visualize cytoskeletal F-actin and nuclei, respectively. Similar to the primary canine tumors, murine primary cell lines expressed a broad suite of Ephs and ephrins at varying levels and with varying intensity and subcellular localization, including several that localized to the nucleus of some or all cell lines.

**Fig. 3.**
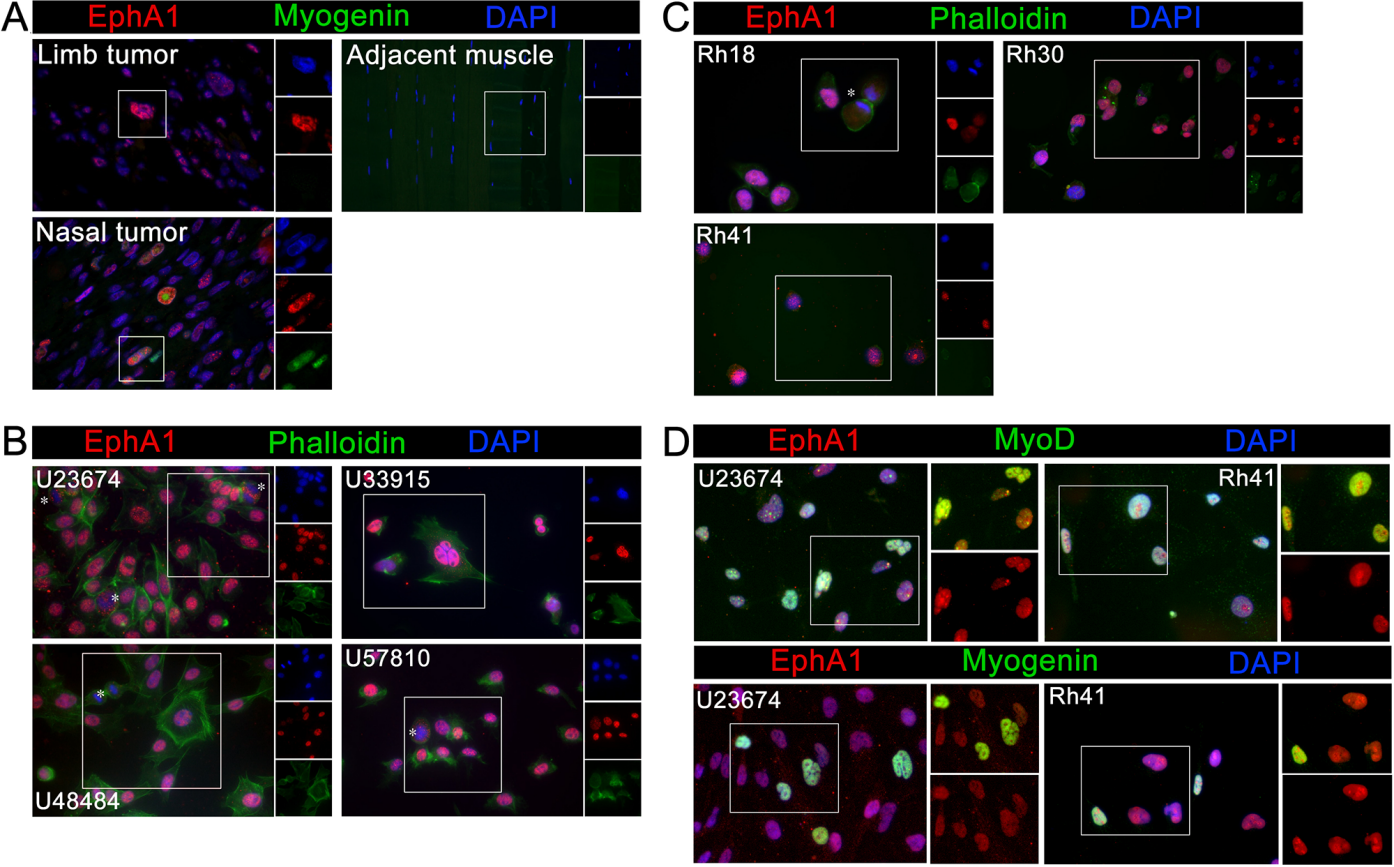
**EphA1 is localized to the nucleus of canine, mouse, and human RMS cells.** Sections of formalin fixed, paraffin embedded primary canine tumors (as in Fig. 1) stained for EphA1 (Alexa 555, red), myogenin (Alexa 488, green) and nuclei (DAPI, blue). EphA1 was expressed in the nuclei of cells from both tumors (A), and myogenin (a marker of RMS) was expressed in some cells of the tumor arising in the nasal cavity. The specimen of the tumor arising from the limb included adjacent healthy skeletal muscle, allowing a direct comparison with the tumor. Neither EphA1 nor myogenin was detected in the adjacent muscle. Four primary mouse RMS cell lines [U23674 (ARMS), U33915 (ERMS), U48484 (ARMS), U57810 (ERMS)] (B) and three human RMS cell lines [Rh18 (ERMS), Rh30 (ARMS), Rh41 (ARMS)] (C) were stained for EphA1 (Alexa 555, red) as well as phalloidin (Alexa 488, green) and DAPI (blue) to visualize cytoskeletal F-actin and nuclei, respectively. EphA1 was expressed in the nucleus of the majority of mouse and human cells assayed; cells lacking strong nuclear localization (marked with asterisks) appear to be undergoing mitosis. The majority of cells in both murine and human RMS cell lines express the myogenic transcription factor MyoD (D), and a subset of cells in each express the related protein myogenin, which would mark terminally differentiated, nonproliferating myocytes in normal muscle but can be expressed by proliferating RMS cells.

The mouse RMS cell lines expressed all of the Ephs and ephrins that were tested. The robustness and localization of the protein expression varied between receptors as well as between cell lines. Many of the Ephs and ephrins exhibited localization patterns consistent with membrane localization (Fig. 2). We also detected Ephs and ephrins in atypical locations, such as EphA8 and ephrin-A2, which showed perinuclear localization. Several of the Ephs and ephrins could be detected in the nucleus as well. Nuclear localization varied between cell lines – for example, EphA5 was detected in the nucleus of U23674 and U57810 cell lines but less strongly in the nucleus of U33915 and U48484 cell lines (Fig. 2). Of the Ephs and ephrins exhibiting nuclear localization, EphA1 was the only that appeared to be exclusively expressed in the nucleus of all four cell lines (Fig. 2 and Fig. 3B).

While RMS is the most common soft tissue tumor in children, it is still a relatively rare cancer with only 350 new cases diagnosed every year in the USA (Gurney et al., 1999). This adds a significant obstacle to obtaining human primary tumor samples, so most molecular analyses are performed in three cell lines amplified from RMS tumors. Rh18 is an ERMS cell line developed from a tumor originating in the perineum, Rh30 is an ARMS cell line developed from a tumor originating in the posterior fossa, and Rh41 is an ARMS cell line from a lung tumor (Houghton et al., 1982; Douglass et al., 1987; Hinson et al., 2013). When cells from all three lines were grown on coverslips and stained for EphA1 expression, all three exhibited nuclear localization of EphA1 (Fig. 3C). As noted in canine primary tumors and mouse cell lines, nuclear EphA1 staining is often punctate (Fig. 3A,B). Interestingly, colocalization with DAPI was lost in dividing cells (Fig. 3, asterisks) and is instead detected throughout the cell.

### EphA1 is localized differentially in distinct phases of the cell cycle

**Fig. 4.**
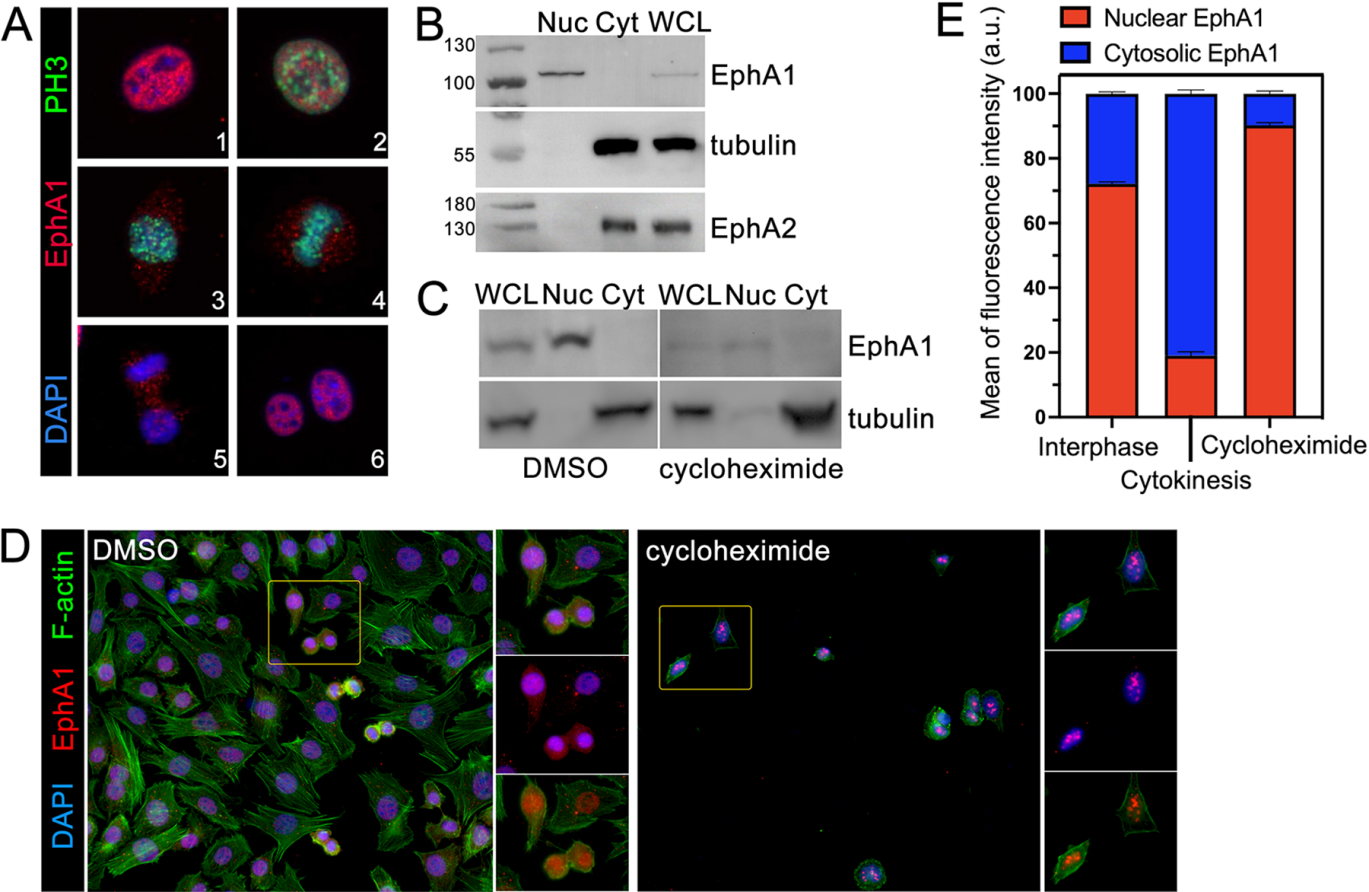
**Full-length EphA1 protein is localized to the nucleus of RMS cells in a cell-cycle-dependent manner.** U23674 cells were stained for EphA1 (Alexa 555, red) and phosphorylated histone 3 (PH3) (Alexa 488, green) to determine the localization of EphA1 throughout the cell cycle; DNA was visualized with DAPI (blue) (A). Cells in interphase (1) and prophase (2) show nuclear localization of EphA1. Cells in prometaphase (3) have diffuse EphA1 staining which is no longer colocalized with DAPI. This localization pattern is maintained through metaphase (4), telophase, and cytokinesis (5), then EphA1 is again restricted to the nucleus after the cell cycle has completed (6). Nuclear and cytoplasmic fractions as well as whole cell lysate of a mouse RMS cell line (U23674) were separated by SDS-PAGE and blotted for EphA1, tubulin (a cytosolic protein), and EphA2 (a membrane protein) (B). EphA1 protein has a predicted molecular weight of 108 kDa, thus the apparent mobility of the EphA1 band detected in WCL and the nuclear fraction suggests that EphA1 is in the nucleus as a full-length receptor. Treatment with the translation inhibitor cycloheximide decreased EphA1 levels by 70%, but EphA1 remained localized to the nucleus by Western blot (C) and immunocytochemistry (D). Quantitation of nuclear vs cytoplasmic EphA1 fluorescence intensity (E) in untreated cells scored as being either in interphase or cytokinesis based on PH3 expression, as well as cycloheximide-treated cells, is in agreement with western blot data.

To investigate if EphA1 localization is correlated with specific phases of the cell cycle, we co-stained the mouse cell line U23674 for EphA1 and phospho-histone H3 (PH3), which is differentially localized at each phase of the cell cycle. Histone H3 serine 10 phosphorylation is high from prophase through metaphase but drops during anaphase and is barely detectable or absent during telophase (Sauvé et al., 1999). When RMS cells first enter the cell cycle in prophase, as indicated by positive PH3 staining, EphA1 colocalizes with DNA indicating that it is still restricted to the nucleus (Fig. 4A, panel 2). Shortly after prophase but before metaphase, EphA1 is no longer colocalized with DNA and instead is detected diffusely throughout the cell (Fig. 4A, panel 3). This diffuse localization is maintained through the rest of mitosis and cytokinesis (Fig. 4A, panels 4/5). Following exit from mitosis, indicated by negative PH3 staining, EphA1 is again restricted to the nucleus and colocalizes with DAPI (Fig. 4A, panel 6).

### EphA1 exists in the nucleus as a full-length polypeptide

The portion of an RTK that is trafficked to the nucleus can have implications for its function, as evidenced by the ErbB family. When full-length ErbB members are trafficked to the nucleus, they have pro-tumorigenic roles including increased cell proliferation, angiogenesis, and metastatic potential mediated through regulating gene expression and interactions with other nuclear proteins (Hynes and Lane, 2005; Jones, 2008; Stephenson et al., 2012). If instead an intracellular domain cleavage fragment is trafficked to the nucleus, it has anti-tumorigenic roles associated with growth suppression through indirect transcriptional regulation (Carpenter and Liao, 2009; Stephenson et al., 2012). Our staining data localizes EphA1 to the nucleus, but because the antibody which detected the protein is raised to the intracellular, C-terminal domain of the protein we could not use immunocytochemistry to differentiate between full-length and cleaved or truncated protein. To ask if EphA1 is in the nucleus as an intracellular fragment or a full-length receptor, we isolated nuclear and cytoplasmic fractions as well as whole cell lysate from mouse ARMS cell line U23674 and performed western blots. The EphA1 band detected in the nuclear fraction and whole cell lysate have the same apparent molecular weight (108 kD), suggesting that EphA1 in the nucleus is a full-length polypeptide rather than a smaller fragment (Fig. 4B).

### Cell-cycle dependent nuclear localization is most likely due to active trafficking

To further investigate the cell-cycle dependence of nuclear EphA1 localization, we treated U23674 cells with cycloheximide to inhibit *de novo* protein translation and arrest the cells at the G2/M boundary (Lockhead et al., 2020). After 36 h of treatment, EphA1 levels were decreased by an average of 70% compared to DMSO-treated cells by western blot (Fig. 4C) but remained in the nuclear fraction. Similar results were obtained when treated cells were stained for EphA1 and PH3 (Fig. 4D), and when total fluorescence of EphA1-stained cells was quantified with respect to nuclear versus cytoplasmic localization (Fig. 4E). These data are consistent with loss of EphA1 nuclear localization during nuclear envelope breakdown prior to mitosis and re-uptake during its re-establishment following mitosis, but do not rule out the possibility of degradation and *de novo* synthesis as an additional mechanism.

## DISCUSSION

Abnormal localization of RTKs to the nucleus occurs in the context of several different types of tumor and is generally associated with progression of the transformed phenotype or protection from cellular gatekeepers. RTK families that have been shown to translocate to the nucleus include ErbB2 in breast cancer, FGFR1 in medulloblastoma, and VEGFR1 in lymphoma (Huo et al., 2014). This study adds EphA1 to the list of RTKs detected in the nucleus of transformed cells. We saw nuclear expression in RMS of three species: canine, mouse, and human. While the mouse cell lines were derived from transgenic mice with RMS-causing mutations, the replication of our results in human patient-derived cell lines and canine primary tumor samples indicates that nuclear localization of EphA1 is a broad property of RMS cells. In addition, the canine limb tumor was imbedded in skeletal muscle, allowing us to compare expression of EphA1 in RMS and skeletal muscle directly. EphA1 was not detected in surrounding skeletal muscle, indicating that the expression and atypical localization is unique to RMS and represents a difference between normal and tumor tissues. EphA1 expression has been detected in mouse primary satellite cells: 50% of mouse satellite cells expression EphA1 at the cell surface (Stark et al., 2011). This is different from the canine tissue, but EphA1 expression in mouse RMS cells is still increased and localized to the nucleus rather than the plasma membrane. In addition, the conserved atypical localization across the three species suggests that it could have conserved roles in RMS formation and/or progression.

To predict what activity nuclear EphA1 may have in RMS, it is useful to compare it with the best-understood instance of nuclear RTK activity in cancer. In the ErbB family, the localization of different fragments of the RTK to the nucleus has distinct and opposite effects: full-length ErbB receptors in the nucleus are tumorigenic, but cleavage fragments are anti-tumorigenic. Because our data indicate that EphA1 is in the nucleus as a full-length protein, if it acts similarly to nuclear ErbB we would expect EphA1 to promote RMS formation or progression. The mechanism of full-length nuclear ErbB translocation is well described: after activation with EGF, it is endocytosed into vesicles where it associates with importin-β. Then it travels back through the Golgi and endoplasmic reticulum to the nuclear membrane, where it travels through nuclear pores and is released from the membrane into the nucleoplasm by Sec61 (Wang et al., 2010). Three other Eph receptors have recently been detected in the nuclei of cancer cell lines: EphA4 was detected in the nucleus of an osteosarcoma cell line, but its function is not known (Kuroda et al., 2008); EphA5 was detected in the nucleus of lung cancer cell lines in response to ionizing radiation (IR), where it binds to ATM to repair DNA damage, preventing IR induced cell death (Staquicini et al., 2015); and EphB4 was detected in the nucleus of prostate cancer cell lines, where it binds to DNA and alters transcriptional levels of genes associated with more advanced prostate cancer (Mertens-Walker et al., 2015). Both EphA5 and EphB4 are present in the nucleus as full-length receptors (Mertens-Walker et al., 2015; Staquicini et al., 2015). While the trafficking mechanism of EphA5 is not known, EphB4 has a bipartite nuclear localization sequence (Mertens-Walker et al., 2015), similar to what we identified in EphA1, and is translocated into the nucleus with importin-α. However, EphA5 and EphB4 are present at both the plasma membrane and the cytoplasm of lung cancer and prostate cancer cells, respectively. Because we did not observe EphA1 in the cytoplasm or at the plasma membrane in nondividing cells, the mechanism by which it is localized to the nucleus appears to diverge from both ErbB and other Ephs. One potential alternate mechanism is based on the requirement that transmembrane proteins such as RTKs be glycosylated during translation at the endoplasmic reticulum prior to trafficking to the plasma membrane (Silhavy et al., 1983).

EphA1 is the first nuclear RTK to be identified in RMS, and RMS is the first cellular context in which EphA1 has been detected in the nucleus. While the mechanism of its nuclear import and subsequent role in RMS have not yet been identified, the addition of EphA1 to the class of nuclear RTKs and its identification in RMS are important advances in both fields.

## MATERIALS AND METHODS

### Cell culture and samples

Mouse RMS cell lines were provided by Dr. Charles Keller. These cell lines were generated from tumors in transgenic mice with the following mutations to cause RMS: U23674 (Myf6Cre/Pax3-Foxo1/p53^−/−^), U33915 (Pax7CreER, Ptch1, p53^−/−^), U48484 (Myf6Cre/Pax3-Foxo1/p53^−/−^), U57810 (Myf6Cre/p53^−/−^) (Keller and Capecchi, 2005). Mouse RMS cell lines are cultured in DMEM with 10% Fetal Bovine Serum (Clontech) and 1% penicillin/streptomycin (Sigma-Aldrich) at 37°C and 5% CO_2_ in a humidified incubator. Human RMS cell lines Rh18, Rh30, and Rh41 were acquired from the Children's Oncology Group at Texas Tech University. Human cell lines were grown in Iscove's Modified Dulbecco's Medium plus 20% Fetal Bovine Serum, 4 mM L-glutamine, 1xITS, and 1% penicillin/streptomycin at 37°C and 5% CO_2_ in a humidified incubator.

### Cycloheximide treatment

U23674 cells were treated with 40 μg cycloheximide in DMSO (Thermo Fisher Scientific, catalogue number 357420050) in normal growth medium (DMEM 1X, 10% FBS, 0.1% gentamicin) for 36 h. Control cells were incubated with equal volume of DMSO in normal growth medium.

### Immunofluorescence

For fluorescent immunocytochemistry of cultured RMS cells, mouse cell lines were plated onto glass coverslips coated with 6.6% gelatin and allowed to adhere at least overnight. Human RMS cell lines were plated onto glass coverslips coated with poly-L-lysine. Coverslips were fixed in cold 4% paraformaldehyde on ice. Cells were blocked in 10% normal goat serum with 1% Nonidet-P40 for 1 h at room temperature for all primaries except for those raised in goat, in which case cells were blocked with 20% BlokHen (Aves Labs) for 1 h at room temperature. Cells were then incubated with primary antibody overnight at 4°C, washed, incubated with secondary antibody for 1 h at room temperature, and washed again. Cells were then stained with Alexa 488-labeled phalloidin (Invitrogen) diluted 1:20 from a stock solution of 6.6 µM into PBS and incubated at room temperature for 30 min. Cells were then washed and mounted in Vectashield containing DAPI (Vector Labs).

Canine tumor specimens isolated from tumors located in the nasal cavity and the limb of two different dogs were donated by Dr. Jeffrey Bryan at the University of Missouri School of Veterinary Medicine, MO, USA. Tumors were formalin fixed and paraffin embedded before sectioning. Sections were deparaffinized in xylene-substituted Histo-Clear (National Diagnostics) using three changes for 5 min each. Sections were then rehydrated by washing in 100% ethanol 2×10 min then 95% ethanol for 10 min followed by a 1-min wash in deionized water with stirring. For antigen retrieval, cells were rinsed in PBS 3×5 min then rinsed in 10 mM citrate buffer for 5 min. Citrate buffer was replaced, and the sections were processed in an Antigen Retriever (Aptum Biologics). After the retrieval cycle finished and sections had cooled, they were rinsed three times with deionized water then PBS for 15 min. After this, sections were blocked and stained as described above for coverslips.

For fluorescence intensity quantification, ImageJ tools ‘ROI manager’ and ‘measure’ were used to compare EphA1 fluorescence intensity in nuclei (ROI based on DAPI) and cytoplasm/membrane (ROI based on phalloidin). For each condition, 10 cells were identified for the analysis: (1) cells in interphase in the untreated cell sample; (2) cells in cytokinesis in the untreated sample; (3) cells in the synchronized (cycloheximide treated) sample. Total values of cytosol and nucleus fluorescence intensities were calculated for each cell and then individual values for cytosol and nucleus for each cell were divided by the total fluorescence value and multiplied by 100.

Primary antibodies (Santa Cruz, unless otherwise noted) and dilutions were as follows: EPHA1 1:200, N-terminal EPHA1 1:100, EPHA2 1:200, EPHA3 1:200, EPHA4 1:200, EPHA7 1:300, EPHA8 1:200, EPHB1 1:200, EPHB2 1:200, EPHB4 1:200, EPHB6 1:200 (AbCam), ephrin-A1 1:200, ephrin-A2 1:200, ephrin-A3 1:200, ephrin-A4 1:200, ephrin-A5 1:200 (AbCam), ephrin-B1 1:200, ephrin-B3 1:300, PH3 1:100. Secondary antibodies were raised in goat and conjugated with Alexa fluorophores and used at 1:500 (Invitrogen). All images were acquired and processed on an Olympus BX61 upright microscope using Slidebook software (Intelligent Imagine Innovations) or µManager software (www.micro-manager.org). Digital background subtraction was performed to remove to remove signal that was less than or equal to levels present in control samples (processed in parallel but without primary antibody) and was applied equally to the entire field.

### Cell fractionation

Subcellular fractionation to isolate nuclei from the cytoplasm was performed based on a previously published method (Suzuki et al., 2010). RMS mouse cell lines were grown to near confluency, washed twice in cold DPBS, and collected in 1 ml of cold DPBS using a plastic cell scraper. Samples were briefly spun for 10 s in microcentrifuge then the supernatant was removed and cells were resuspended in 900 µl of ice-cold 0.1% NP40 in PBS and triturated five times with a P1000 micropipette. 300 µl was removed to be the ‘whole cell lysate’ and stored on ice. Leftover sample was briefly spun for 10 s. 300 µl was removed to be the ‘cytosolic fraction’ and stored on ice. Remaining supernatant was removed and pellet was resuspended in 900 µl ice-cold 0.1% NP40. Samples were briefly spun for 10 s and supernatant was discarded. Pellet was resuspended in 135 µl ice-cold 0.1% NP40 to be the ‘nuclear fraction’ and stored on ice. Roche protease inhibitor cocktail (11836170001) was added to each of the samples and the nuclear fraction and whole cell lysate were passed through a 30-gauge needle (BD PrecisionGlide) to rupture nuclei. All lysates were stored at −80°C until needed.

### Western blot

6× Laemmli buffer [60 mM 1.5 M Tris-Cl pH 6.8 (Thermo Fisher Scientific), 10% glycerol (Thermo Fisher Scientific), 2% sodium dodecyl sulfate (Thermo Fisher Scientific), 5% β-mercaptoethanol, 0.01% bromophenol blue (JT Baker)] was added to fractionated U23674 cell lysates equivalent to 2×10^5^ cells and boiled for 10 min. Samples and ladder (PageRuler Prestained Protein Ladder, Thermo Fisher Scientific) were run on a 10% polyacrylamide gel at 130 V then transferred to a PVDF membrane. Membranes were blocked for 1 h at room temperature in StartingBlock (TBS) Blocking Buffer (Thermo Fisher Scientific) while rolling. Primary antibody was diluted in StartingBlock and incubated with membrane overnight while rolling at 4°C. Membranes were then washed three times in TBST (19 mM Tris pH 7.4 (Thermo Fisher Scientific), 137 mM NaCl (Thermo Fisher Scientific), 2.7 mM KCl (Thermo Fisher Scientific), 0.05% Tween-20 (Sigma-Aldrich) for 20 min each at room temperature. Membranes were then incubated in secondary antibody diluted in StartingBlock for 1 h at room temperature with rolling then washed three times in TBST for 20 min with rolling. Bands were visualized with SuperSignal West Femto Maximum Sensitivity Substrate (Thermo Fisher Scientific) and detected using a LAS3000 imager (Fujifilm). Primary antibodies and dilutions used were EphA1 1:1000 (Santa Cruz), EphA2 1:1000 (Santa Cruz), tubulin 1:1000 (Chemicon), and phosphotyrosine 1:1000 (Upstate). Secondary antibodies used were goat anti-rabbit HRP 1:50,000 (Santa Cruz) and goat anti-rat HRP 1:10,000 (Pierce).
